# The role of interleukin (IL)-23 in regulating pain in arthritis

**DOI:** 10.1186/s13075-022-02777-y

**Published:** 2022-04-25

**Authors:** Kevin M.-C. Lee, Jonathan P. Sherlock, John A. Hamilton

**Affiliations:** 1 The University of Melbourne, Department of Medicine, Royal Melbourne Hospital, Parkville, Victoria Australia; 2 Janssen Research and Development LLC, Spring House, PA USA; 3 Kennedy Institute of Rheumatology, University of Oxford, Oxford, UK; 4 Australian Institute for Musculoskeletal Science (AIMSS), The University of Melbourne and Western Health, St. Albans, Victoria Australia

**Keywords:** IL-23, Arthritis, Pain and innate immunity

## Abstract

Current understanding of IL-23 biology, with its link to other pro-inflammatory cytokines, for example, IL-17 and granulocyte macrophage-colony stimulating factor (GM-CSF), is primarily focused on T lymphocyte-mediated inflammation/autoimmunity. Pain is a significant symptom associated with many musculoskeletal conditions leading to functional impairment and poor quality of life. While the role of IL-23 in arthritis has been studied in mouse models of adaptive immune-mediated arthritis using targeted approaches (e.g., monoclonal antibody (mAb) neutralization), the literature on IL-23 and arthritis pain is limited. Encouragingly, the anti-IL-23p19 mAb, guselkumab, reduces pain in psoriatic arthritis patients. Recent evidence has suggested a new biology for IL-23, whereby IL-23 is required in models of innate immune-mediated arthritis and its associated pain with its action being linked to a GM-CSF-dependent pathway (the so-called GM-CSF➔CCL17 pathway). This Commentary discusses the current understanding of potential cytokine networks involving IL-23 in arthritis pain and provides a rationale for future clinical studies targeting IL-23p19 in arthritis pain.

## Arthritis and pain

Arthritic diseases, such as psoriatic arthritis (PsA) and rheumatoid arthritis (RA), are chronic inflammatory diseases, which impact patients both physically and psychologically. Neuropathic-like pain as evidence of abnormal pain processing is common in such patients [1, 2] and one of their highest priorities is chronic pain relief.

Nociception (pain) is the process by which chemical, mechanical or thermal stimuli are detected by specialized peripheral neurons called nociceptors [3]. During inflammation, the threshold for nociceptor neurons to fire action potentials is reduced by the triggering of key receptors, such as transient receptor potential vanilloid subfamily member 1 (TRPV1) and the sodium ion channel, Na_v_1.8 [4], leading to pain sensitivity or “hyperalgesia.” Studies are elucidating the role of specific immune cells and mediators in controlling pain sensitivity in different disease contexts. For example, in antigen-induced arthritis (AIA), macrophages have been observed to infiltrate both peripheral tissue (i.e., joints) and the dorsal root ganglion (DRG) [5, 6], and secreted cytokines, such as tumor necrosis factor (TNF), granulocyte macrophage-colony stimulating factor (GM-CSF) and CCL17, have been associated with inflammatory and arthritic pain development [5, 7]. Conversely, monocytes and macrophages can contribute to the resolution of inflammatory pain via a mechanism that is dependent on IL-10 signaling in DRGs [8, 9].

In this Commentary, we will focus on the role of IL-23 in regulating arthritis pain.

## IL-23 in arthritis

IL-23 was discovered when a search for IL-6 cytokine family members identified the novel protein subunit, “p19” [10]. This protein is poorly secreted from cells but, when bound to the p40 subunit of IL-12, forms the secreted and bioactive cytokine, IL-23. A key role for IL-23 is to stimulate production of IL-17 from memory T cells [11], which were later termed Th17 cells [12]. While IL-23 acts late on adaptive T cells, it can act rapidly and directly on IL-23R-expressing innate-like lymphocytes, such as type 3 innate lymphoid cells [13].

IL-23p19 gene-deficient (*Il23p19*^*-/-*^) mice are protected from the development of collagen-induced arthritis (CIA) [14] and antigen-induced arthritis (AIA) [15]. Blocking IL-23 activity, using a neutralizing anti-IL-23p19 mAb following immunization [16, 17], but before disease onset, suppressed the severity of CIA [16]. In contrast, administration of the anti-IL-23p19 mAb following the first clinical signs of CIA gave no improvement [16]. These data suggest that IL-23 is required for disease onset but not for the effector phase of arthritis. There are clinical trial data indicating that anti-IL-23p19 mAb treatment met the primary endpoint (i.e., American College of Rheumatology 20% improvement) in PsA patients [18–20] but not in RA patients [21].

## IL-23 and arthritis pain

Little is known about the role(s) of IL-23 in pathological pain development. However, it has been found in clinical trials that PsA patients receiving guselkumab (CNTO 1959, Janssen), a neutralizing mAb to IL-23p19, achieved both minimal disease activity, a composite index that includes the patient’s assessment of pain visual analog scale, and also significant improvements in the SF-36 physical component score, an assessment that includes bodily pain [18–20].

As regards experimental arthritis pain, it was reported recently, using the T cell-independent zymosan-induced arthritis (ZIA) and zymosan-induced paw inflammation models, that *Il23p19*^*-/-*^ mice were protected from developing arthritis and inflammatory pain (i.e., weight bearing deficit), respectively [22]. Furthermore, it was found that *Il23p19*^*-/-*^ mice were protected from GM-CSF-, TNF-, and CCL17-driven arthritis pain and disease [22], with these models also being T cell independent [23, 24]. Mechanistically, such protection in *Il23p19*^*-/-*^ mice, at least when studied in the ZIA model, was found to correlate with reduced *Csf2* (the gene encoding GM-CSF) and *Ccl17* mRNA, but not *Tnf* mRNA, expression. Interestingly, in the ZIA joints, *Il23p19* mRNA expression was found to be dependent on GM-CSF and TNF, but not on CCL17 [22]. These data suggest that the requirement for IL-23 in arthritis pain is associated with these inflammatory cytokines, with the responding cell(s) and/or the cellular source of IL-23 not being an adaptive T cell population(s). Conversely, direct injection of IL-23 in the plantar region induces inflammatory pain that also requires these cytokines as well as cyclooxygenase (COX) activity [22]. These findings provide the first evidence that the contribution of IL-23 to arthritis and inflammatory pain has potential links to TNF, GM-CSF, CCL17, and eicosanoid function. However, precisely how IL-23 contributes to arthritis pain development requires further study.

There are other mechanistic studies exploring how IL-23 can regulate pain. IL-23/IL-23 receptor (IL-23R) signaling in astrocytes has been implicated in central neuropathic pain in a model of sciatic nerve injury, and interaction between IL-23, CX3CL1, and IL-18 in the spinal cord was proposed [25]; also, IL-23-regulated T cell-derived cytokines, including possibly IL-17A, contribute to the inflammatory response in another model of neuropathic pain [26]. Interestingly, nociceptive sensory neurons can interact with dermal dendritic cells (DCs) to drive IL-23-mediated psoriasiform skin inflammation and resistance to cutaneous candidiasis [27, 28]. There is evidence for a link between the biologies of IL-23 and neuropeptides/neurotrophins, such as nerve growth factor (NGF) [29], calcitonin gene-related peptide (CGRP) [27, 28, 30] and substance P [31–33], all of which can be important mediators in pain development in humans [34] and have been implicated in inflammatory diseases of the skin (see, for example [35]). A recent study has demonstrated that IL-23 and IL-17A drive the crosstalk between immune cells (i.e., macrophages) and neurons for mechanical pain induction [36]. Additionally, cyclooxygenase products, such as prostaglandin E_2_, have been linked to IL-23 biology (see, for example, [37–44]).

## IL-23 and arthritis pain: questions and issues

While there is some literature on the role of IL-23 in arthritis pain, several questions and issues which need to be addressed are as follows.

As mentioned, there are clinical trial data indicating that IL-23 blockade is effective in treating PsA [18–20], but not RA [21]. It would be interesting to know for which other arthritis patients IL-23 is important for their pain (and disease) and whether early and/or late IL-23p19 targeting would be effective. Also, there needs to be more research and clinical data on whether the beneficial effects of IL-23 blockade on pain are dependent or not on its effects on local inflammation.

There is evidence that pathological changes in the CNS, such as infiltration of immune cells, are also crucial components for maintaining chronic arthritis pain [6]. Although IL-23 biology is often associated with that of T lymphocytes in inflammation/autoimmunity, as outlined above, a recent study has demonstrated that IL-23 is required for different inflammatory arthritis pain models that exhibit lymphocyte-independent biology [22]. Little is known regarding the signficance of the role of lymphocyte-independent IL-23 biology in general, as well as for arthritis pain progression. More information is needed on which cell type(s) responds to IL-23 and which cell type(s) functions as its source. One possible responding cell type could be synovial fibroblasts as they have been shown to express IL-23R, and their activation by IL-23 can lead to TNF production [45].

For its involvement in arthritis pain, it is not known whether IL-23 can act peripherally and/or centrally. The current data on the effectiveness of systemic anti-IL-23p19 mAb administration in the control of arthritis pain [22] suggest perhaps that IL-23 is acting peripherally in the particular model studied. Given that IL-23 expression can be detected in DRGs [25, 46], it would be of interest to explore whether and, if so, how IL-23 can contribute to the activation of nociceptors for arthritis pain development.

We mentioned above that, in a recent study, IL-23 has been linked to the inflammatory cytokines/chemokines, TNF, GM-CSF, and CCL17, for the development of arthritis pain [22]. In a nerve injury model, an interaction between IL-23 and other cytokines/chemokines has been proposed [25], although the nature of these links is unknown. Which additional cytokines/chemokines may be critically linked with IL-23 in the regulation of arthritis pain are unknown. It is possible that there might not be a simple linear sequence of cytokine production and activity, but instead perhaps multiple mediator loops contributing to arthritis pain development [22]. It was also reported that neuropeptides/neurotrophins, namely NGF, CGRP, and substance P, are required for GM-CSF- and CCL17-driven inflammatory pain [47]. These mediators have been linked elsewhere with IL-23 biology [27–33] and exploring their link with IL-23 in arthritis pain would be worthwhile. The importance of other mediators (e.g., COX metabolites) in the action of IL-23 in arthritis pain remains to be determined.

This Commentary has focused mainly on IL-23 and its regulation of arthritis pain. How significant IL-23 generally is for the control of pain (and itch [48]) and how relevant are the IL-23-dependent mechanisms in arthritis pain to other conditions where pain is a debilitating symptom remain open areas for investigation. As an example, perhaps IL-23 may be contributing to the frequently reported abdominal pain in inflammatory bowel disease patients [49].

## Conclusion

In contrast to the literature on lymphocyte-dependent IL-23 biology, it was recently reported that IL-23 is involved in innate immune-driven arthritis pain and disease with its links to other inflammatory cytokines, namely GM-CSF, CCL17, and TNF [22]. In this Commentary, we have mainly focused on the current understanding of the role of IL-23 in arthritis pain and the current evidence supporting its targeting for treating such pain. We have also listed a number of outstanding questions and issues that need to be addressed in order to advance our understanding of the role of IL-23 in arthritis pain.

## Data Availability

Not applicable.

## Abbreviations

IL-23: Interleukin-23
PsA: Psoriatic arthritis
RA: Rheumatoid arthritis
TRPV1: Transient receptor potential vanilloid subfamily member 1
AIA: Antigen-induced arthritis
GM-CSF: Granulocyte macrophage-colony stimulating factor
TNF: Tumor necrosis factor
CIA: Collagen-induced arthritis
NGF: Nerve growth factor
CGRP: Calcitonin gene-related peptide
ZIA: Zymosan-induced arthritis
COX: Cyclooxygenase
DRG: Dorsal root ganglion
IL-23R: IL-23 receptor
DCs: Dendritic cells
mAb: Monoclonal antibody
